# Evaluation of molybdenum recovery from sulfur removed spent catalyst using leaching and solvent extraction

**DOI:** 10.1038/s41598-020-58972-x

**Published:** 2020-02-06

**Authors:** Debabrata Pradhan, Dong-Jin Kim, Lala Behari Sukla, Archana Pattanaik, Seoung-Won Lee

**Affiliations:** 1 Biofuels and Bioprocessing Research Center, ITER, Siksha ‘O’ Anusandhan (Deemed to be University), Bhubaneswar, 751030 India; 2 Mineral Resources Research Division, Korea Institute of Geoscience and Mineral Resources (KIGAM), Daejeon, 305350 South Korea; 3 Nano Engineering Division, School of Engineering, Chungnam National University, Daejeon, 305764 South Korea

**Keywords:** Pollution remediation, Chemical engineering

## Abstract

In this article a new spent catalyst sample preparation method was implemented for the purpose of molybdenum leaching in a single step. Further molybdenum and vanadium in the leach liquor were separated and their concentrations were enriched using the solvent extraction and stripping techniques. The impervious sulfur (S^0^) layer of the spent catalyst sample was removed using carbon disulfide (CS_2_). The advantages of S^0^removal were evaluated by conducting different sets of the Mo leaching experiments and they were further examined by varying different conditions such as three lixiviants, hydrogen peroxide (H_2_O_2_) addition, and three leaching parameters. The leaching rate increased in an order, e.g. acetone washed < acetone-CS_2_ washed < acetone washed-H_2_O_2_ < acetone-CS_2_ washed-H_2_O_2_, for the experimental concentration range of different lixiviants with the maximum of 94.8%(w/w) Mo dissolution in a single step. Optimization of the pulp density was important as the interaction of lixiviant molecules with multiple reacting solid particles decreased the driving force of the chemical reactions. The solvent extraction followed by the stripping technique was found to be excellent as the concentration of vanadium and molybdenum enriched from 0.55 to 1.9 M and 0.0448 to 1.08 M, respectively.

## Introduction

Molybdenum (Mo) and vanadium (V), and their products are used in different applications like catalysis, alloying, aerospace industries, electrical connections, industrial motors, and filaments^1–3^. Generally they are extracted from their respective primary ores^1,2^. However, their reclamation from different spent catalysts (SCs) has been examined using hydrometallurgical and pyro-hydrometallurgical techniques^4–7^. There are different techniques, such as roasting followed by leaching, baking in acid followed by leaching and direct leaching, have been employed for their recovery^8–20^. Also bioleaching has been tried^21–30^. However, simultaneous leaching of the metals like Ni, V, and Mo has always been difficult due their individual chemical identity in the aqueous medium^6,9,18,21,24^. Therefore, a multi-step or sequential leaching process has been applied for their leaching^6,18^.

**Figure 1:**
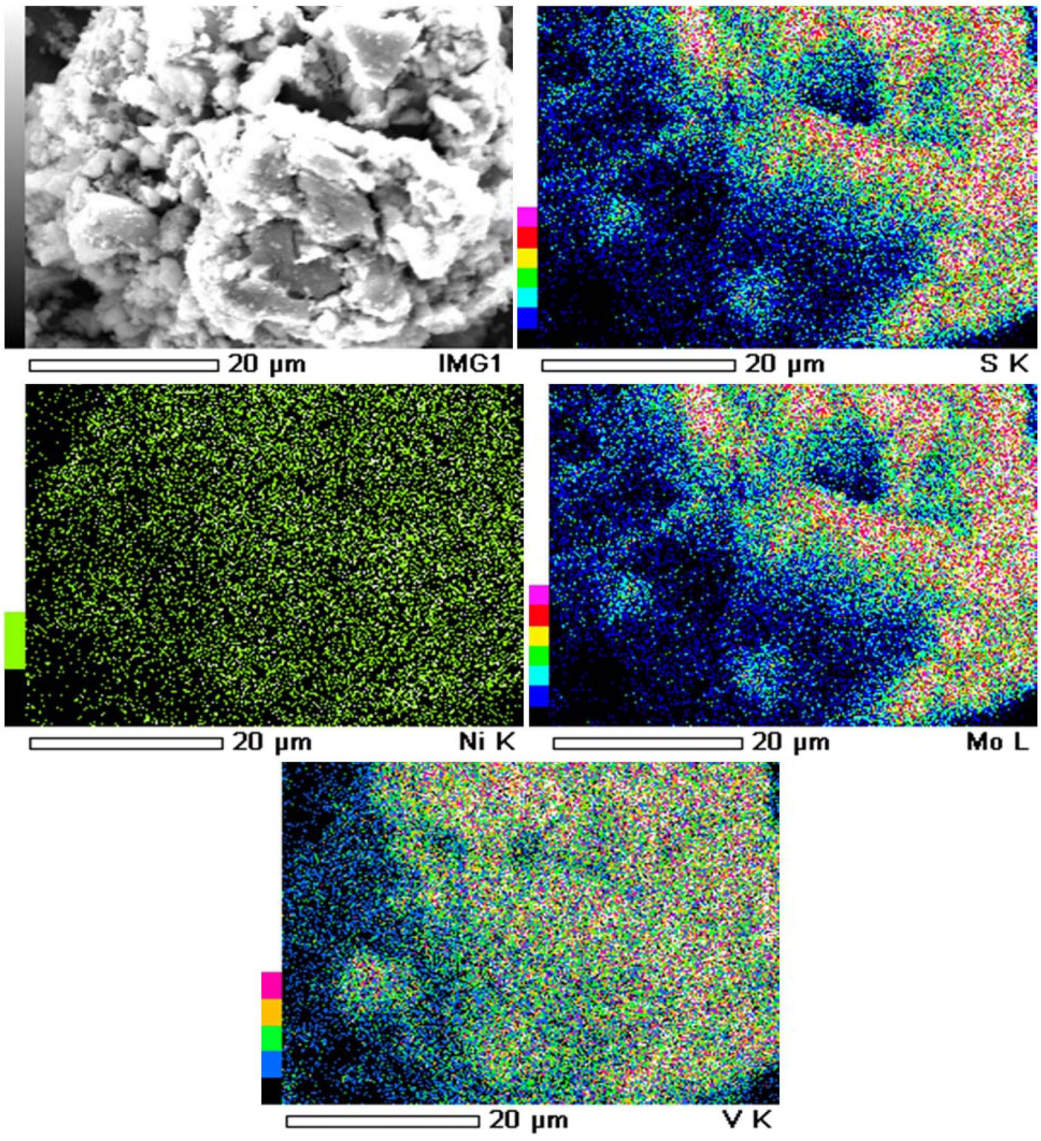
EDX analysis of spent catalyst.

The sequential leaching is not a low-cost process as it undergoes different sub-processes and develops complexity during scale-up. Assembling the sub-processes is a headache during the pilot scale designing. Mishra *et al*.^31^ and Kim *et al*.^32^ have examined the problems associated with the SCs that lead to the sequential leaching, and found that it is Mo-matrix. The XRD analysis has revealed the presence of both sulfide and oxide form of Mo^32^. Olson and Clark^33^ have shown the refractory nature of Mo-sulfide which leads to its lower dissolution. The EDX analysis of the particular SC under this investigation is shown in Fig. 1 which shows that the association of S^0^ with the Mo-matrix within the SC matrix^32^. Since the S^0^ is a hydrophobic substance, it impedes the contact between Mo-matrix and lixiviants during the leaching^31^. Hence the mutual effect of the refractoriness of Mo-sulfide and the impervious S^0^ layer leads to the lower Mo dissolution^31–34^.

The impervious S^0^ layer should be removed in order to build the contact between Mo-matrix and lixiviant during the dissolution of Mo from the SC. It can be removed by dissolving in a suitable solvent^31,35^. Austin *et al*.^35^ has used three solvents such as liquid SO_2_, CS_2_, and CCl_4_ to determine the solubility of S^0^. They have found that the S^0^ dissolving capacity of SO_2_, CS_2_, and CCl_4_ are 0.015, 28.45 and 1.25%(w/v), respectively. Since the solubility of S^0^ is higher in CS_2_, Mishra *et al*.^31^ and Kim *et al*.^32^ have used it as the solvent to remove the S^0^ present in SC, though they have not studied its advantage on Mo leaching in details.

Issues related to the Mo leaching from a SC sample are sorted out on the basis of leaching experiments in this paper. The main objective of experiments is the complete Mo dissolution in a single step instead of a multi-step leaching process by addressing the obstructions. The extraction, separation, and concentration enrichment of Mo and V from the leach liquor is reported. Finally a process flow-diagram is suggested.

## Experimental

### Chemicals

Only analytical grade chemicals and deionized water were used in the experiments, unless otherwise specified.

### Pretreatment of SC

The SC sample was obtained from the SK Incheon Petrochem, South Korea and used as the metallic resource for the leaching experiments. The as such SC accumulated different crude oil impurities and organic pollutants on its surface possibly during the refinery process. Since its surface contents are hydrophobic in nature, they should be washed before employing it in the leaching experiments. The boiled acetone was chosen over all other organic solvents for the washing^30^. The acetone washing of the SC was performed in a soxhlet for 2 hr. It was observed that the oily surface washed away completely during this washing period. Then the acetone washed SC (ASC) was removed from the soxhlet and first dried in the open air followed by drying in a hot oven at 50 °C until a constant mass obtained. The dried ASC was ground to powder using a porcelain mortar and pestle, and then sieved in to different size fractions. The particle size distribution (−106 + 45 µm)of the ASC powder was chosen for the leaching experiments as it was found suitable^21^. This specific sized ASC powder was stored in a desiccator and further used in the leaching studies.

### Presence of S^0^

**Figure 2:**
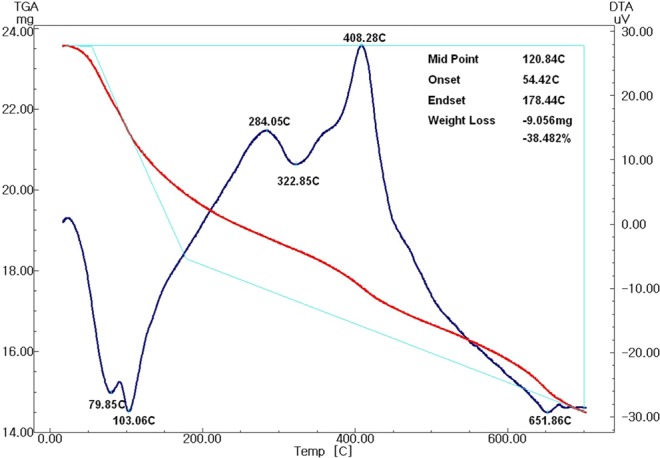
TG-DTA analysis of ASC sample.

Previously our group has shown the association of S^0^ and Mo-matrix within the SC particle^32^. Further, its presence was confirmed by analyzing the thermo gravimetric analysis/differential scanning calorimetry (TG-DTA/DSC) analysis of the ASC using TG-DTA instrument (SHIMADZU). The temperature was increased from 20 to 700 °C with a linear heating rate of 10 °C/min during the analysis. Figure 2 shows the TG-DTA result of the ASC. There are total of five peaks in the Fig. 2. The exothermic peaks at 284 and 408 °C may be due to the loss of hydrocarbon and the transformation of sulfides to oxides of metals, respectively. The three endothermic peaks may correspond to the S^0^. The endothermic peaks at 79, 103 and 322 °C may be due to the α → β transition of S^0^, the melting of β-S^0^ and the complete sublimation of S^0 ^^36^.

### CS_2_ washing of SC

The solubility S^0^ is 241.6 g/L in CS_2_ at room temperature^35^. Therefore, the S^0^ was removed from the ASC sample using CS_2_ as the solvent at room temperature. For this purpose 100 g ASC was added to a beaker containing 500 mL CS_2_ and stirred gently with the help of an overhead stirrer for 30 min. Then the slurry was filtered through the Whatman filter paper and the residue was collected. The residue was then dried in the open air followed by in a hot air oven at 50 °C until a constant mass obtained. The residue was calledacetone + CS_2_ washed SC (ACD) sample which was stored in a desiccator and further used in the leaching experiments. The metal content of the ACD sample was analyzed by following the standard acid digestion process and ICP analysis. Previously our group has reported the metal content in the SC after different pretreatment methods^30^. The metal content of the ACD sample was further added to that table and is shown in Table 1.

**Table 1:** Composition of SC after different pretreatment processes(in g/kg).

SC sample specification	Al	V	Ni	Mo	S
Raw SC	142	78	17	12	106
Acetone washed SC	195	90	20	13.8	115
Acetone + CS_2_ washed SC	206	96	21.4	14.8	58
Raw SCcalcination at 700 °C	204	98	22	14	21
Acetone washed SCcalcination at 700 °C	208	101	22	14	18

### Leaching

The leaching experiments were conducted in 250 mL conical flask containing 100 mL of lixiviants. The Mo leaching of both ASC and ACD was carried out using three different leaching reagents such as H_2_SO_4_, Na_2_CO_3_, and NaOH. Previously leaching of different SC samples was conducted using the above leaching reagents^31,34^. However, leaching of the SC samples after the removal of S^0^ was not reported in details. Further, the two-step leaching process was applied and reported^31,34^. Since this manuscript describes the improvement of Mo leaching rate after the S^0^ removal, the comparison of its dissolution was described on the basis of the above leaching mediums. The SC contains both sulfide and oxide of Mo. Direct dissolution of the Mo-oxides takes place in different acid and alkali solutions, however, the Mo-sulfides require an oxidant for the oxidation of S^2−^ to S^6+^ with simultaneous dissolution of Mo. So 10 mL of H_2_O_2_ was added to different leaching reagents maintaining the lixiviant volume 100 mL. On the H_2_O_2_ aid, it further splitted to another two more sets, such as ASC + H_2_O_2_(AHP) and ACD + H_2_O_2_(ACHP). As a result four different SC conditions, such as ASC, AHP, ACD and ACHP, were developed for three lixiviants. The leaching reactions are shown in Eqs. 1–6 ^4–6,31,34^. The experiments were conducted for 120 min. The time was counted after starting the agitation of content at 300 rpm with the help of a magnetic stirrer, which followed the addition of specified SC sample into the lixiviant. The experiments were conducted at ambient temperature i.e. 25 °C. The concentration of lixiviants and the pulp density were maintained as 1.0 M and 30%(w/v), respectively, unless and otherwise specified. For the analysis of metal concentration in the leach liquors, samples were collected in every 10 min interval and prepared with following precautions. About 2 mL of slurry was withdrawn from the running experiment using a 10 mL rubber head glass dropper without stopping the agitation and transferred into a 2 mL microcentrifuge tube, and then centrifuged using a microcentrifuge. Then exact 1 mL of supernatant was pipetted out using a micropipette and transferred into a 100 mL volumetric flask. Further it was acidified with 20 mL of 10%(v/v) HCl and the volume made up to mark by adding deionized water. The samples were analyzed by ICP-AES (JOBIN-YVON,JY38) after required dilutions.

1 $${{\rm{MoO}}}_{3}+{{\rm{H}}}_{2}{{\rm{SO}}}_{4}\to {{\rm{MoO}}}_{2}{{\rm{SO}}}_{4}+{{\rm{H}}}_{2}{\rm{O}}$$

2 $${{\rm{MoO}}}_{3}+2{\rm{NaOH}}\to {{\rm{Na}}}_{2}{{\rm{MoO}}}_{4}+{{\rm{H}}}_{2}{\rm{O}}$$

3 $${{\rm{MoO}}}_{3}+{{\rm{Na}}}_{2}{{\rm{CO}}}_{3}\to {{\rm{Na}}}_{2}{{\rm{MoO}}}_{4}+{{\rm{CO}}}_{2}$$

4 $${{\rm{MoS}}}_{2}+9{{\rm{H}}}_{2}{{\rm{O}}}_{2}\,({\rm{in}}\,{{\rm{H}}}_{2}{{\rm{SO}}}_{4})\to {{\rm{MoO}}}_{2}{{\rm{SO}}}_{4}+{{\rm{H}}}_{2}{{\rm{SO}}}_{4}+8{{\rm{H}}}_{2}{\rm{O}}$$

5 $${{\rm{MoS}}}_{2}+6{\rm{NaOH}}+6{{\rm{H}}}_{2}{{\rm{O}}}_{2}\to {{\rm{Na}}}_{2}{{\rm{MoO}}}_{4}+2{{\rm{Na}}}_{2}{{\rm{SO}}}_{4}+6{{\rm{H}}}_{2}{\rm{O}}$$

6 $${{\rm{MoS}}}_{2}+3{{\rm{Na}}}_{2}{{\rm{CO}}}_{3}+9{{\rm{H}}}_{2}{{\rm{O}}}_{2}\to {{\rm{Na}}}_{2}{{\rm{MoO}}}_{4}+2{{\rm{Na}}}_{2}{{\rm{SO}}}_{4}+9{{\rm{H}}}_{2}{\rm{O}}+3{{\rm{CO}}}_{2}$$

The leaching rate was calculated using the formulae given in Eq. 7.

7 $${\rm{Y}}=\frac{{{\rm{C}}}_{{\rm{L}}}}{{\rm{X}}\times {\rm{PD}}\,}$$

where,

Y = Leaching rate in %(w/w)

C_L_ = Concentration of metal in leach liquor, mg/L

X = Metal content in the SC, %(w/w)

PD = Pulp density, weight of SC (in g) in 100 mL leaching media.

### Solvent extraction

Solvent extraction (SX) experiments were conducted for a synthetic leach liquor whose concentration based on the composition of leach liquor obtained from the leaching condition (e.g. ACHP; NaOH, 2.0 M; pulp density, 30%(w/v)). The concentrations of nickel (Ni), V and Mo in the leach liquor were found to be 0, 0.554 and 0.0439 M, respectively. The concentration of leach liquor gives the representative solution for the SX experiments. Since the above mentioned condition is optimum for the leaching experiments, the representative solution for the SX experiments were prepared based on the concentration of leach liquor obtained from the above condition. Therefore, the composition of synthetic solution was maintained at 0.55 and 0.045 M for V and Mo, respectively, by adding appropriate amount of NaVO_3_ and Na_2_MoO_4_.2H_2_O to the deionized water. The pH of synthetic solution was maintained at 9.6 by adding NaOH solution, as it was the final pH of the original leach liquor. The quaternary amine salt such as Aliquat 336 diluted in toluene was used as the organic phase for the SX^7^. Aliquat 336 is a basic extractant suitable for the anionic loading. The SX experiments were conducted using the 60 mL separatory funnels containing the total volume of 24 mL for 30 min of agitation using a centrifugal shaker in ambient temperature. The volumetric ratio of aqueous to organic phase (A:O) were defined by keeping the total volume of the content 24 mL.The SX conditions were varied as specified. After completion of each SX run exactly 1 mL of sample was withdrawn from the aqueous phase and transferred into a 100 mL volumetric flask. The samples were prepared and analyzed accordingly (section 2.5). Followed by the SX, required stripping experiments were conducted using different solutions as specified. The stripping conditions and sampling were mostly similar to the SX experiments.

All the experimental runs were triplicated and the standard deviations of the results were within ±3%.

## Results and Discussions

### Effect of contact time and lixiviants

**Figure 3:**
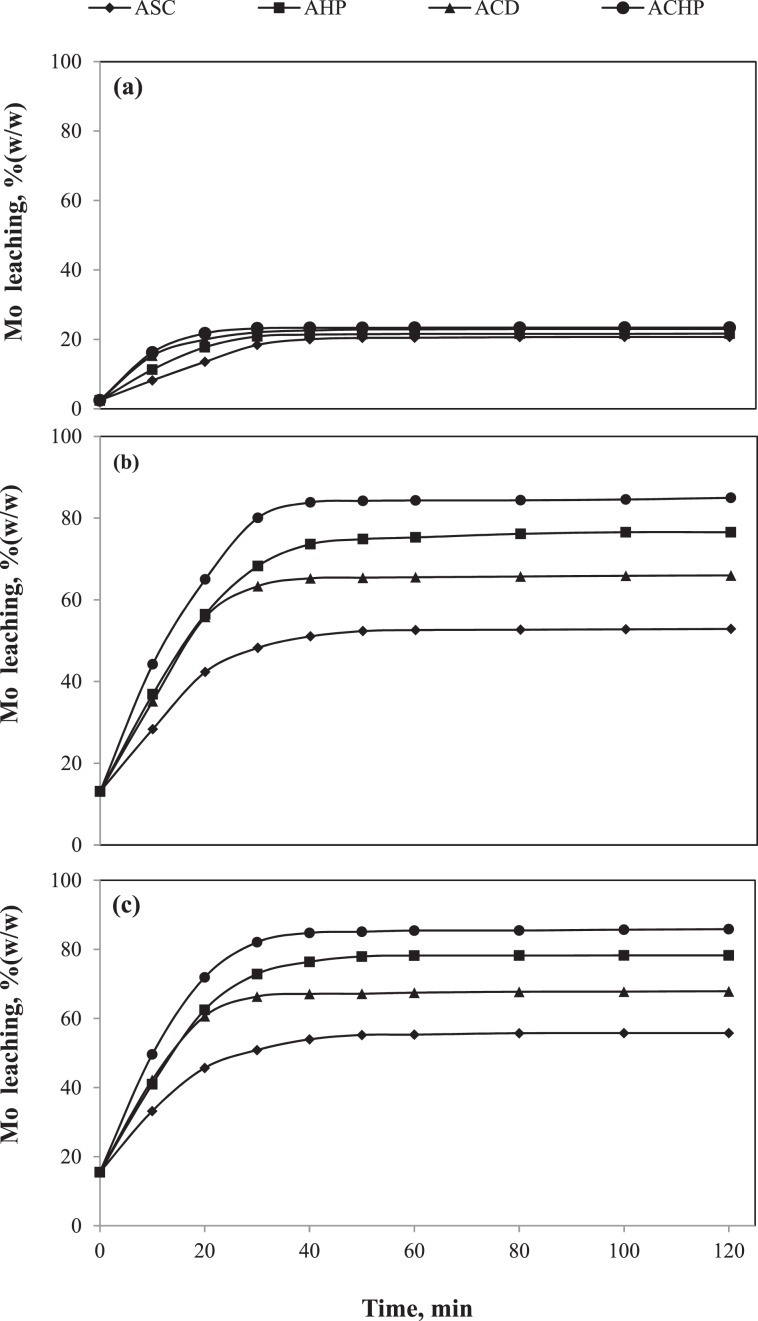
Effect of contact time on Mo dissolution for different SC conditions: (**a**) 1.0 M H_2_SO_4_; (**b**) 1.0 M Na_2_CO_3_; (**c**) 1.0 M NaOH.

Acid leaching of Mo from four SC conditions, such as ASC, AHP, ACD and ACHP, was conducted using 1.0 M H_2_SO_4_ at different time interval and is shown in Fig. 3(a). Its leaching rate for the SC conditions did not show substantial deviation. The Mo forms either molybdic sulfate (MoO_2_SO_4_) or molybdic acid (H_2_MoO_4_) in the acidic medium. Also the MoO_4_^2−^ ion polymerizes in the acidic^37^. The solubility of metallic salts/ions formed in the leach liquor limits the leaching rate. Since different salts/ions of Mo were less soluble in the acidic medium, the H_2_SO_4_ leaching did not show the effective Mo dissolution^37^. The leaching rates for the SC conditions were just above 20%(w/w) before reaching the equilibrium. The maximum leaching rate and the corresponding equilibrium time for all set of experiments were evaluated and are given in Table 2. The equilibrium time of ASC was 40 min, and that for AHP, ACD and ACHP were 30 min. The lesser equilibrium time proved that those conditions favored the Mo dissolution; however, the solubility controlled the leaching rate. Since Mo is highly soluble in the alkali medium, its leaching was conducted for the SC conditions in the 1.0 M Na_2_CO_3_ medium. Figure 3(b) shows the effect of contact time on the Mo dissolution in the Na_2_CO_3_ medium. From Fig. 3(b), it can be observed that the leaching rate increased quickly for both ACD and ACHP within 20 min compared to their respective without CS_2_ washed conditions such as ASC and AHP. In the case of ASC and AHP, the impervious S^0^ was present in the Mo-matrix which prevented the contact between Mo-matrix and lixiviants resulting in the slower Mo diffusion^31^. When the impervious S^0^ was washed away in the case of ACD and ACHP, the sulfur voids developed within the SC particles which made the easy penetration of the lixiviants through the voids and they reacted with the underlying Mo-matrix^32^. The leaching rate and equilibrium time were 52.6%(w/w) and 50 min, respectively, in the case of ASC which may be due to dissolution of Mo-oxide easily approachable at the surface of SC particles. When the leaching rate of the ACD case compared to that of the ASC, it increased up to 65.2%(w/w) and the equilibrium time decreased to 40 min which were possible due to the S^0^ removal resulting in the easy approach of the lixiviants. In the case of AHP, the leaching rate and equilibrium time respectively increased up to 76.1%(w/w) and 80 min which may be due to the oxidation of both Mo-sulfide and S^0^^34^. This was further confirmed by the leaching of ACHP condition. In the case of ACHP, the leaching rate increased up to 84.2%(w/w) and the equilibrium time decreased to 50 min, which were due to the mutual effect of the easy penetration of lixiviants in to the underlying Mo-matrix and the oxidation of Mo-sulfide present in the core of SC particles^32,34^. When the Na_2_CO_3_ leaching data compared to that of H_2_SO_4_ leaching, it was observed that the Mo leaching rate was much higher for the SC conditions which was possible due to the thermodynamic stability of Mo ions in the alkaline medium^37^. Although the Mo leaching rate increased when the lixiviant was switched from H_2_SO_4_ to Na_2_CO_3_, the complete Mo leaching was yet to be achieved. Therefore, further Mo leaching experiments were conducted using 1.0MNaOHfor the SC conditions. The effect of contact time on the Mo leaching rate using NaOH medium is shown in Fig. 3(c). The leaching rate in NaOH followed a similar contact time frame to that of NaCO_3_; however, the leaching rate increased by 2 to 4%(w/w) for different SC conditions. From Table 2, it can be observed that the equilibrium time for ASC, ACD and ACHP were same as those of Na_2_CO_3_ as the lixiviant, however, it decreased from 80 to 60 min in the case of AHP which proved that the oxidizing environment was more favoured by NaOH than NaCO_3_. Still the maximum leaching rate did not reach 90%(w/w) in the most favorable condition i.e. ACHP + 1.0 M NaOH. This may be due to the depletion of attacking species in the leaching medium for the complete Mo dissolution^32^. Therefore, further concentration of the lixiviants was varied keeping other parameters constant.

**Table 2:** Leaching rate of Mo and corresponding equilibrium for different SC conditions.

Leaching specification	SC condition	Leaching rate, %(w/w)	Equilibrium time, min
H_2_SO_4_, 1 M	ASC	20.41	40
	AHP	21.35	30
	ACD	22.86	30
	ACHP	23.32	30
Na_2_CO_3_, 1 M	ASC	52.61	50
	AHP	76.16	80
	ACD	65.27	40
	ACHP	84.21	50
NaOH, 1 M	ASC	55.22	50
	AHP	78.21	60
	ACD	67.11	40
	ACHP	85.11	50

### Effect of lixiviant concentration

In order to evaluate the effect of lixiviant concentration on the Mo leaching rate, the concentration of H_2_SO_4_, Na_2_CO_3_, and NaOH was varied as 0.1, 0.5, 1.0, 1.5 and 2.0 M. The leaching rates using different concentration of lixiviants are shown in Table 3. The variation of leaching rate for the SC conditions was noticeable at the lower concentration of H_2_SO_4_; however, it was not substantial for further study (Table 3**)**. It was only 10.4%(w/w) in the case of ASC in 0.1 M H_2_SO_4_ and increased by just above 5%(w/w) in other three cases (e.g.AHP, ACD and ACHP). With the increase of H_2_SO_4_ concentration, there could hardly be any difference observed as the saturation of Mo in the acidic leach liquor. However, its difference was observed in the alkali leaching as it increased linearly with the increase of Na_2_CO_3_ concentration from 0.1 to 1.0 M (Table 3). Its increment was substantial from 1.0 to 1.5 M and on further increase from 1.5 to 2.0 M there could hardly any increase. With the increase of NaOH concentration 0.1 to 2.0 M for the SC conditions, it increased constantly. For a certain concentration of either Na_2_CO_3_ or NaOH its increment followed an order i.e. ASC < ACD < AHP < ACHP; however, the order was more contrast at higher concentration. It achieved the maximum of 94.88%(w/w) when the ACHP reacted with 2.0 M NaOH.

**Table 3:** Effect of lixiviants concentration on Mo leaching rate.

	ASC	AHP	ACD	ACHP
**H**_**2**_**SO**_**4**_	**%(w/w) of leaching**			
0.1 M	10.41	15.47	16.86	16.99
0.5 M	18.94	20.54	22.55	22.56
1.0 M	20.68	21.67	22.99	23.39
1.5 M	23.26	23.84	23.86	24.69
2.0 M	24.59	24.84	24.17	24.82
**Na**_**2**_**CO**_**3**_	**%(w/w) of leaching**			
0.1 M	20.59	30.63	26.75	34.24
0.5 M	38.26	55.76	49.65	58.53
1.0 M	52.87	76.56	65.98	84.97
1.5 M	55.33	78.86	68.63	86.36
2.0 M	56.25	78.87	70.54	86.83
**NaOH**	**%(w/w) of leaching**			
0.1 M	26.38	34.77	28.26	37.32
0.5 M	40.66	61.87	54.77	64.19
1.0 M	55.78	78.27	67.89	85.85
1.5 M	61.69	84.87	71.38	93.77
2.0 M	62.99	86.86	71.54	94.88

### Effect of pulp density

**Figure 4:**
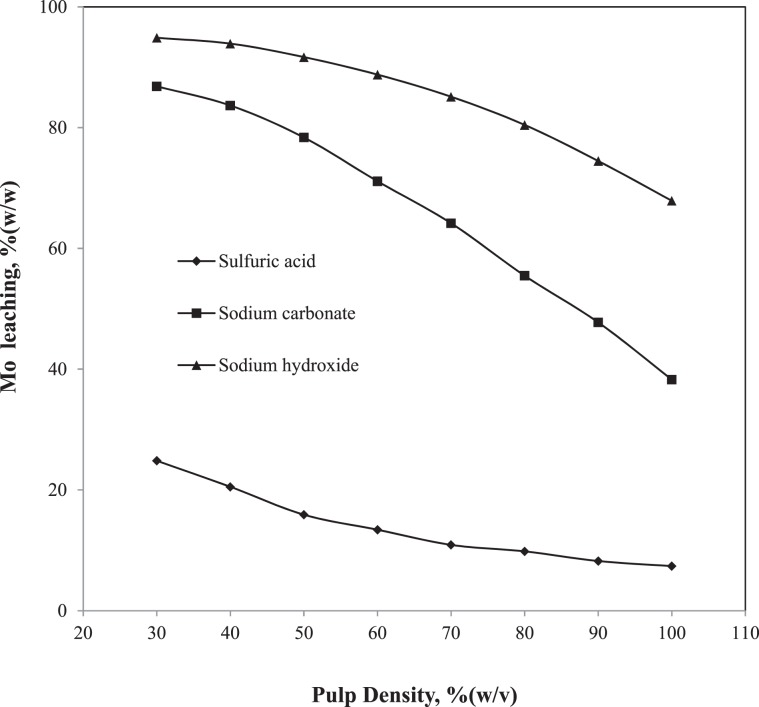
Effect of pulp density on Mo leaching.

The pulp density was maintained at 30%(w/v) for the above studies. In order to evaluate the Mo leaching rate at different pulp densities, further experiments were conducted by varying it as 30, 40, 50, 60, 70, 80, 90 and 100%(w/v). As the ACHP condition showed better leaching rate over other three SC conditions, the pulp density study was conducted for the ACHP using the lixiviants such as H_2_SO_4_, Na_2_CO_3_ and NaOH. However, the concentration of lixiviants increased to 2.0 M in order to maintain the adequate attacking species required for the elevated amount of Mo and other metals at the higher pulp densities. The Mo leaching rate plotted versus pulp density is shown in Fig. 4. The leaching rate decreased with the increase of pulp density for both acidic and alkaline medium which was obvious. Since the concentration of lixiviants was increased to maintain the chemical reaction environment at the higher pulp density, the leaching rate decreased may be due to vulnerable interactions between the reacting SC particles and the lixiviants^38^. The reacting SC particles did not experience any change of the lixiviants concentration at their interface when the lower pulp density was implemented. With the gradual increase of the pulp density, the suspension became more concentrated which made the reaction particles competitive among each other for the lixiviant molecules^38^. As a result it significantly reduced the driving force of the chemical reactions. The degree to which a chemical reaction slowed down by this interaction can be characterized by a dimension less parameter ‘η’ which can be expressed by Eq. 8 as follows^38^:

8 $${\rm{\eta }}=\frac{{\rm{a}}}{{\rm{b}}}\times \frac{{{\rm{VC}}}_{{\rm{l}}}}{{{\rm{M}}}_{{\rm{s}}}}$$

where,

a = number of mole of Mo in the balanced chemical equations (Eqs. 1–6);

b = number of mole of lixiviant in the balanced chemical equations (Eqs. 1–6), (for H_2_SO_4_ and NaOH, b = 1 and for Na_2_CO_3_, b = 2);

V = volume of suspension in L;

C_l_ = initial molar concentration of lixiviant;

M_s_ = initial number of mole of Mo in the SC sample.

**Figure 5:**
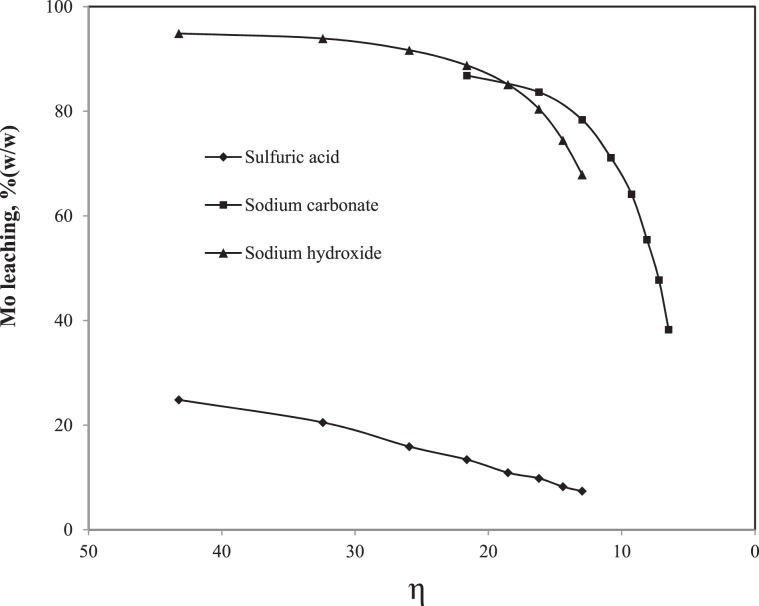
Effect of degree of chemical reaction slowed down (η) on the leaching rate.

The ‘η’ values were calculated from the experimental data and then the leaching rate in %(w/w) was plotted versus it for different lixiviants (Fig. 5). It can be observed that the Mo leaching rate decreased with the decrease of ‘η’ value which was due to the interaction between the lixiviant molecules and the multiple reacting SC particles at the higher pulp density decreased the driving force of the chemical reactions^38^.

### Solvent extraction

#### Variation of pH on SX

**Figure 6:**
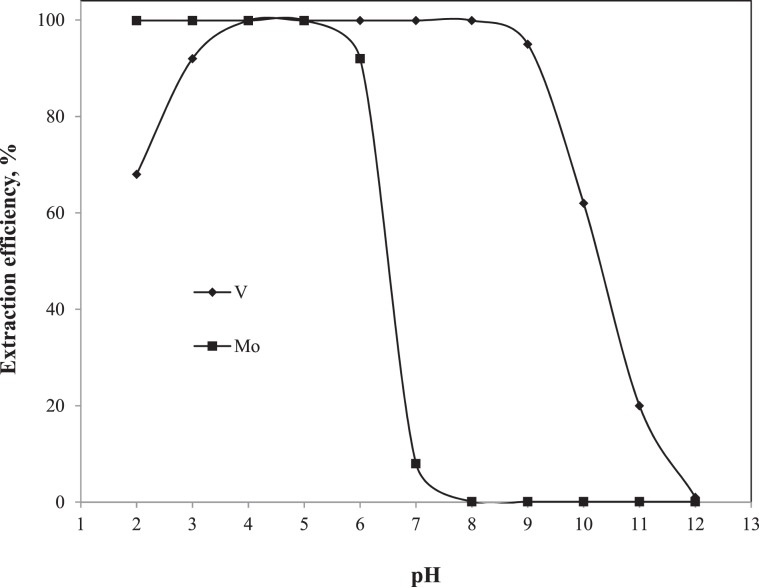
Effect of pH on V and Mo extraction.

The SX of V and Mo from the synthetic leach liquor was conducted using Aliquat 336 diluted in toluene. In order to get the pH extraction isotherm curve, the V and Mo were extracted individually from the pure metallic solution at different pH range using 20%(v/v) Aliquat 336 by keeping the aqueous to organic phase ratio (A:O) constant at 1:1. The concentration of V and Mo were 0.55 and 0.045 M, respectively. Since the leach liquor was alkaline, the pH was varied from 12.0 to 2.0 for both V and Mo solutions by adding either concentrated H_2_SO_4_ or NaOH solution. The SX efficiency of V and Mo were evaluated and plotted versus pH, and the graph is shown in Fig. 6. The Fig. 6 shows that the V extraction increased with the decrease of pH from 12.0 to 8.0 and reached the maximum of 99.9% at pH 8.0. The maximum retained from pH 8.0 to 4.0 and on further decrease of the pH from 4.0 to 2.0 the V extraction decreased. For the Mo extraction, there was negligible extraction observed from pH 12.0 to 8.0. It increased with the decrease of pH from 8.0 to 5.0 and then remained at maximum (99.6%) from pH 5.0 to 2.0. The Fig. 6 shows that both V and Mo co-extracted below pH 8.0. Therefore, it was convenient to extract V at pH > 8.0 in the first step followed by the Mo extraction from the V-barren solution at pH < 5.0 in the second step. This was supported by the separation factor (SF) data given in Table 4. The pH variation data, such as concentration in organic phase, distribution coefficient (D), and SF, are given in Table 4. The maximum SF value of V over Mo was of 998001 at pH 8.0. With the superior SF value chances of the Mo co-extraction could be minimized during the V extraction at pH 8.0. For the remaining SX study, the V was extracted from the mixture solution at pH 8.0 followed by the Mo extraction from the V-barren liquor at pH 5.0.

**Table 4:** Effect of pH variation on SX of V and Mo. (Conditions:Organic phase, 20%(v/v) Aliquat 336; A:O, 1:1; [V], 0.55 M; [Mo], 0.045 M).

pH	Concentration in organic phase, M		Distribution coefficient (D)		Separation factor (SF)	
	V	Mo	V	Mo	V/Mo	Mo/V
2	0.375	0.045	2.14	999.0	0.002	465.8
3	0.508	0.045	12.26	999.0	0.012	82.2
4	0.549	0.045	999.0	999.0	1.0	1.0
5	0.549	0.045	999.0	999.0	1.0	1.0
6	0.549	0.041	999.0	11.82	84.5	0.012
7	0.549	0.004	999.0	0.09	10893.9	91 × 10^−6^
8	0.549	45 × 10^−6^	999.0	10 × 10^−4^	998001.0	1 × 10^−6^
9	0.523	18 × 10^−6^	19.41	4 × 10^−4^	48501.0	20 × 10^−6^
10	0.343	13.5 × 10^−6^	1.65	3 × 10^−4^	5506.7	181 × 10^−6^
11	0.113	9 × 10^−6^	0.26	2 × 10^−4^	1297.0	771 × 10^−6^
12	0.006	4.5 × 10^−6^	0.01	1 × 10^−4^	111.2	8991 × 10^−6^

**Figure 7:**
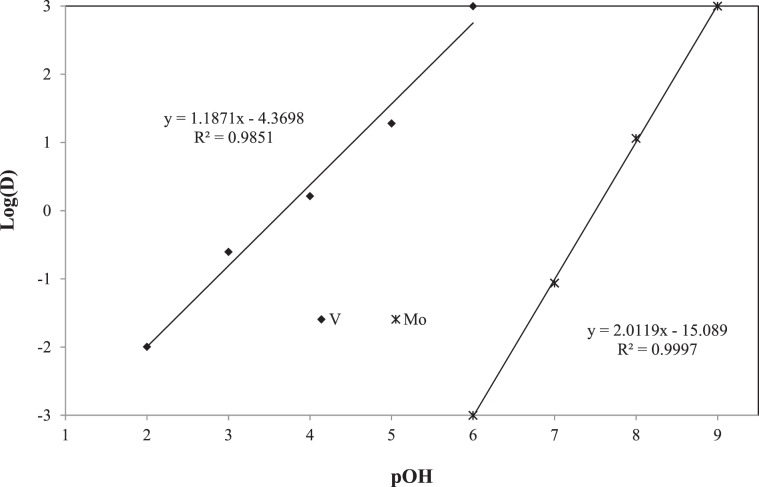
pOH extraction isotherm curve of V and Mo.

The extraction isotherm curves for both V and Mo were developed by plotting log D versus equilibrium pOH, instead of versus pH, using the pH variation data for the better explanation of the equilibrium equations. It is shown in Fig. 7 which shows that the slopes of the curves were found to be 1.187 and 2.011 for V and Mo, respectively^31^. From the values of slopes, the following equilibrium equations are proposed for the SX of V (Eq. 9) and Mo (Eq. 10):

9 $${{\rm{VO}}}_{3({\rm{aq}})}^{-}+{{\rm{L}}}^{+}{{\rm{Cl}}}_{({\rm{org}})}^{-}+{{\rm{NaOH}}}_{({\rm{aq}})}\to {{\rm{L}}}^{+}{{\rm{VO}}}_{3({\rm{org}})}^{-}+{{\rm{NaCl}}}_{({\rm{aq}})}+{{\rm{OH}}}_{({\rm{aq}})}^{-}$$

10 $${{\rm{MoO}}}_{4({\rm{aq}})}^{2-}+2{{\rm{L}}}^{+}{{\rm{Cl}}}_{({\rm{org}})}^{-}+2{{\rm{NaOH}}}_{({\rm{aq}})}\to {({{\rm{L}}}^{+})}_{2}{{\rm{MoO}}}_{4({\rm{org}})}^{2-}+2{{\rm{NaCl}}}_{({\rm{aq}})}+2{{\rm{OH}}}_{({\rm{aq}})}^{-}$$

#### Extraction-stripping circuit of V

**Figure 8:**
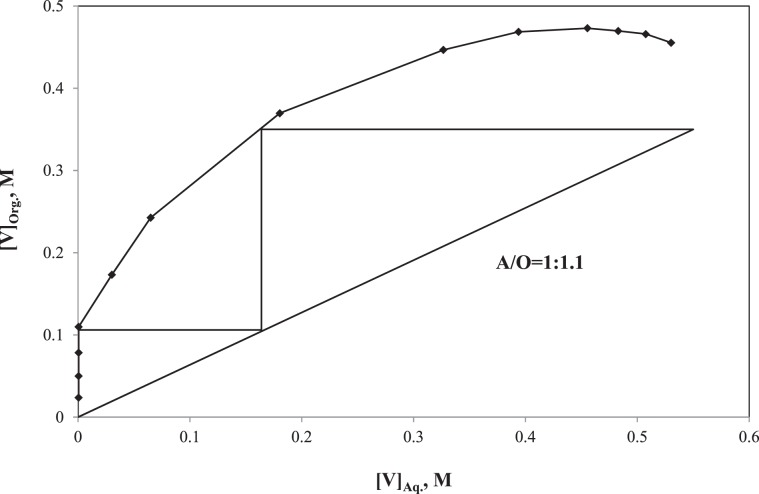
McCabe-Thiele plot of V extraction.

**Figure 9:**
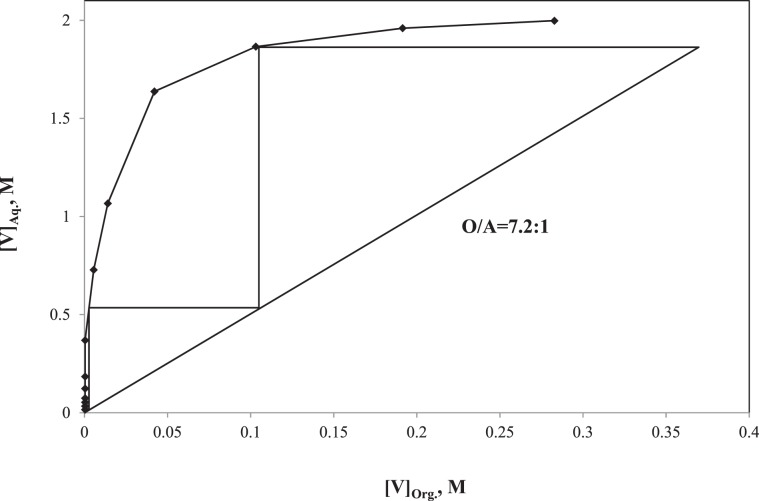
McCabe-Thiele plot of V stripping.

The V extraction isotherm was established by varying the A:O ratio as 1:23, 1:11, 1:7, 1:5, 1:3, 1:2, 1:1, 2:1, 3:1, 5:1, 7:1, 11:1, and 23:1 while other parameters (e.g. total volume, 24 mL; pH, 8.0; organic phase, 5%(v/v) Aliquat 336 in toluene; [V], 0.55 M; [Mo], 0.045 M) kept constant. In order to find out the number of stages required for its extraction, a McCabe-Thiele plot was established using the experimental data and is shown in Fig. 8^39^. For the McCabe-Thiele plot, the operating line was based on the 70% V loading at the A:O ratio of 1:1.1. By putting the operating line, 0.55 M of V can completely be extracted from the leach liquor in a two-stage process. The co-extraction of Mo was very marginal as it was 4 × 10^−5^ M. The stripping of V from the loaded organic was conducted by using three different solutions, such as NaOH (2.0 M), NH_4_OH (2.0 M)and Na_2_CO_3_ (2.0 M), at the O:A ratio and the total volume of 1:1 and 24 mL, respectively. For the stripping experiments, the loaded organic was collected from the SX experiment with A:O of 1:1 where the V and Mo concentration was 0.38 and 4 × 10^−5^ M, respectively. The stripping solutions, such as NaOH, NH_4_OH and Na_2_CO_3_, respectively recovered 99.8, 94.2 and 82.6% of V from the loaded organic. Therefore, further stripping isotherm was evaluated using 2.0 M NaOH solution. In order to evaluate the number of stage required for the V stripping, the O:A ratio were varied as 1:23, 1:11, 1:7, 1:5, 1:3, 1:2, 1:1, 2:1, 3:1, 5:1, 7:1, 11:1 and 23:1while other parameters (e.g. total volume, 24 mL; [NaOH], 2 M; [V], 0.38 M; [Mo], 4 × 10^−5^ M) kept constant. The McCabe-Thiele plot for the V stripping was developed using the O:A variation data where the operating line based on the 70% stripping at the O:A ratio of 7.2:1 and is shown in Fig. 9. By putting the operating line, 0.38 M of V can completely be stripped from the loaded organic in a two-stage process. The V concentration enriched from 0.55 M in the leach liquor to 1.9 M in the stripped solution through the SX followed by the stripping whereas the negligible Mo co-extraction resulted in its concentration 2 × 10^−4^ M in the stripped solution.

#### Extraction-stripping circuit of Mo

**Figure 10:**
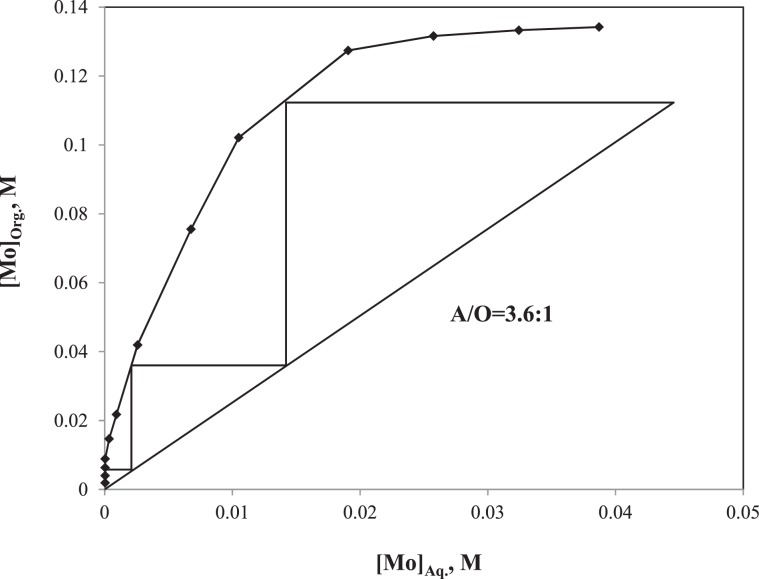
McCabe-Thiele plot of Mo extraction.

**Figure 11:**
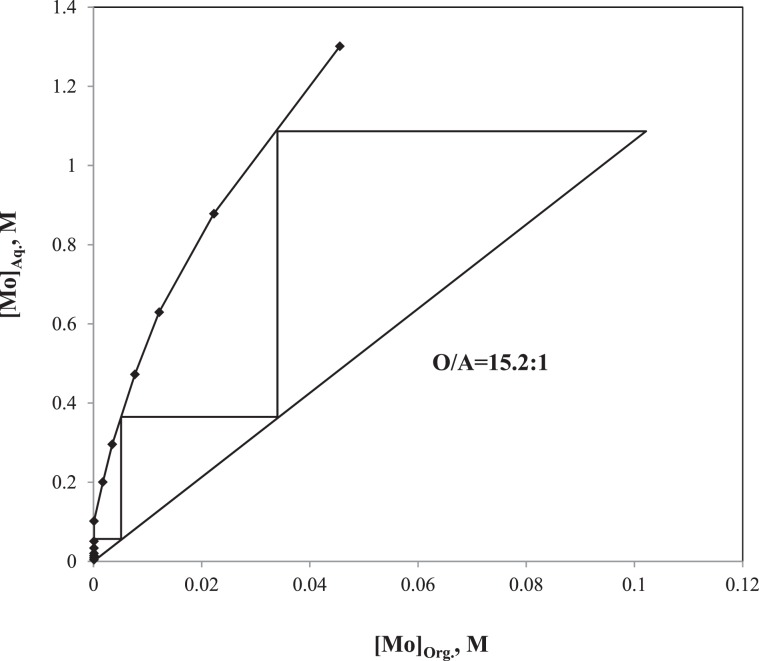
McCabe-Thiele plot of Mo stripping.

After the V recovery at pH 8.0, the concentrations of V and Mo in the raffinate (V-barren leach liquor) were 2 × 10^−5^ and 0.0448 M, respectively. The SX was conducted to recover Mo from the raffinate by varying the A:O ratio as 1:23, 1:11, 1:7, 1:5, 1:3, 1:2, 1:1, 2:1, 3:1, 5:1, 7:1, 11:1 and 23:1 while other parameters (e.g. total volume, 24 mL; pH, 5.0; organic phase, 5%(v/v) Aliquat 336 in toluene; [V], 2 × 10^−5^ M; [Mo], 0.0448 M) kept constant. The McCabe-Thiele plot was developed using the Mo extraction data and is shown in Fig. 10 ^39^. The operating line was based on the 70% Mo extraction at the A:O ratio of 3.6:1. By putting the operating line, the 0.0448 M Mo can completely be extracted from the V-barren leach liquor in a three-stage process. The concentration of V and Mo in the loaded organic were 5 × 10^−5^ and 0.102 M, respectively. The Mo stripping from the loaded organic was conducted by using three different solutions, such as NaOH (2.0 M), NH_4_OH (2.0 M), and Na_2_CO_3_ (2.0 M), similar to the conditions of the V stripping. The NaOH, NH_4_OH and Na_2_CO_3_ respectively recovered 99.9, 96.8 and 92.5% of Mo from the loaded organic. Therefore, further stripping isotherm was evaluated using the 2.0 M NaOH solution. In order to evaluate the number of stage required for the Mo stripping, the O:A ratio were varied as 1:23, 1:11, 1:7, 1:5, 1:3, 1:2, 1:1, 2:1, 3:1, 5:1, 7:1, 11:1 and 23:1 while other parameters (e.g. total volume, 24 mL; [NaOH], 2 M; [V], 5 × 10^−5^ M; [Mo], 0.102 M) kept constant. The McCabe-Thiele plot was developed using the O:A variation data and is shown in Fig. 11. The operating line based on the 70% stripping of Mo from the loaded organic at the O:A ratio of 15.2:1. By putting the operating line, the 0.102 M of Mo can completely be stripped from the loaded organic in a three-stage process. The Mo concentration enriched from 0.0448 M in the V-barren leach liquor to 1.08 M in the stripped solution through the SX followed by the stripping whereas the negligible V co-extraction resulted in its concentration 1.2 × 10^−4^ M in the stripped solution.

**Figure 12:**
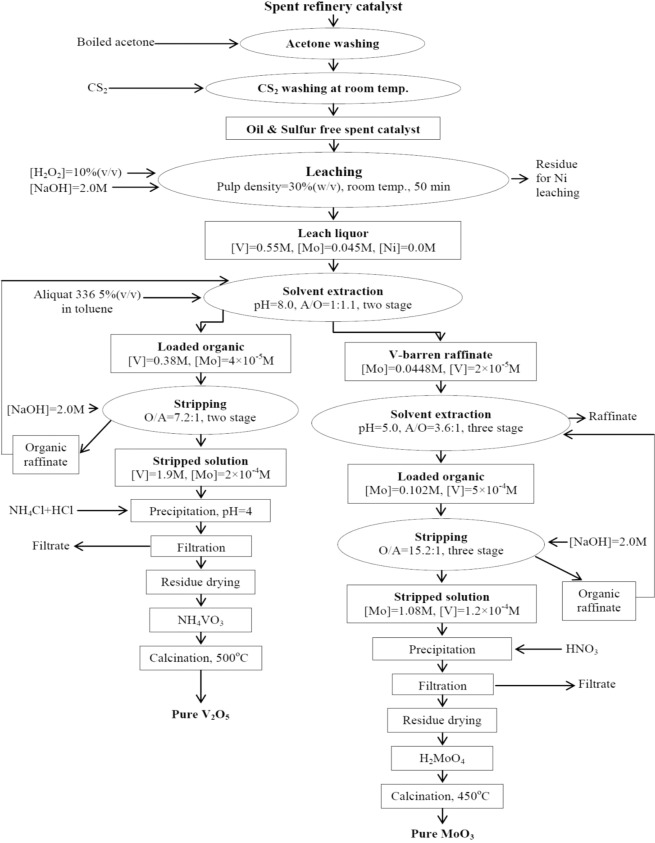
Process flow-sheet^40,41^.

From the above experimental evaluations, a flow-sheet was developed for the recovery of Mo and V from the SC sample and is shown in Fig. 12. The material balances starting from the leaching to stripping are mentioned in the flow-sheet. In the flow-sheet the final precipitation of Mo and V from respective stripped solutions were given on the basis of their standard precipitation processes^40,41^.

## Conclusion

Both sulfide and oxide of Mo were present in Mo-matrix of the SC sample. The chemical properties of Mo and its association with the impervious S^0^ layer within the SC created difficulties during its leaching. The CS_2_ washing process successfully removed the S^0^ from the SC sample. When the impervious S^0^ was washed away, the sulfur voids developed within the SC particles which made the easy penetration of the lixiviants through the voids and they reacted with the underlying Mo-matrix resulting in the higher Mo dissolution. The leaching rate increased in an order, e.g. acetone washed < acetone-CS_2_ washed < acetone washed-H_2_O_2_ < acetone-CS_2_ washed-H_2_O_2_; however, the order was more contrast at the higher concentration of lixiviants. The maximum Mo dissolution was 94.8%(w/w) for the acetone-CS_2_ washed-H_2_O_2_ condition reacted with 2.0 M NaOH. The Mo leaching rate decreased with the increase of pulp density due to the interaction of lixiviant molecules with the multiple reacting solid particles decreased the driving force of the chemical reactions. The SX of V and Mo from the synthetic leach liquor was successful using Aliquat 336 diluted in toluene as the organic phase. The pH variation experiments of SX showed that the V should be extracted from the leach liquor at pH > 8.0 in the first step followed by the Mo extraction from the V-barren solution at pH < 5.0 in the second step. The V could completely be extracted from the leach liquor in a two-stage process using 5%(v/v) Aliquat 336 at the A:O ratio of 1:1.1. The V loaded organic could completely be stripped in a two-stage process using 2.0 M NaOH at the O:A ratio of 7.2:1. The Mo in the V-barren leach liquor could completely be extracted using 5%(v/v) Aliquat 336 in a three-stage process at the A/O ratio of 3.6:1. The Mo loaded organic could completely be stripped in a three-stage process using 2.0 M NaOH at the O:A ratio of 15.2:1. The laboratory scale experiments developed a model for the recovery of V and Mo from SC through the leaching and SX techniques; however, higher scale study should be conducted prior to development of the pilot scale operation.
